# Whole-Genome Sequence Analysis of Multidrug-Resistant Enterobacter hormaechei Isolated from Imported Retail Shrimp

**DOI:** 10.1128/MRA.01103-20

**Published:** 2020-12-10

**Authors:** Nagaraju Indugu, Laxmi Sharma, Charlene R. Jackson, Prashant Singh

**Affiliations:** a Department of Nutrition, Food and Exercise Sciences, Florida State University, Tallahassee, Florida, USA; b Department of Clinical Studies, Center for Animal Health and Productivity, School of Veterinary Medicine, University of Pennsylvania, Kennett Square, Pennsylvania, USA; c Bacterial Epidemiology and Antimicrobial Resistance Research Unit, U.S. Department of Agriculture, Agricultural Research Service, Athens, Georgia, USA; Georgia Institute of Technology

## Abstract

Here, we announce the draft genome sequence of Enterobacter hormaechei 2B-MC1, isolated from a shrimp sample collected from a farmer’s market in Atlanta, Georgia. The assembled genome sequence observed was 4,661,561 bp long with a G+C content of 55.3%. The isolate harbored *sul1*, *sul2*, *qnrA1*, *oqxB*, *dfrA23*, *bla*_ACT_, *floR*, *fosA*, *tet*(A), *aph(6)-Id*, and *aph(3″)-Ib* antibiotic resistance genes.

## Abstract (precis)

Here, we announce the draft genome sequence of Enterobacter hormaechei 2B-MC1, isolated from a shrimp sample collected from a farmer’s market in Atlanta, Georgia. The assembled genome sequence observed was 4,661,561 bp long with a G+C content of 55.3%. The isolate harbored *sul1*, *sul2*, *qnrA1*, *oqxB*, *dfrA23*, *blaACT*, *floR*, *fosA*, *tet*(A), *aph(6)-Id*, and *aph(3″)-Ib* antibiotic resistance genes.

## ANNOUNCEMENT

We isolated and characterized a multidrug-resistant (MDR) Enterobacter hormaechei 2B-MC1 strain from raw farm-raised shrimp from Ecuador which was collected from a farmer’s market in Atlanta, Georgia, on 30 March 2019. Thirty-one shrimp samples were screened for the presence of extended-spectrum β-lactam strains as previously described (1). The 110 isolates obtained were purified by streaking onto nutrient agar. The genus and species of isolates were identified after 16S rRNA gene sequencing using 27F and 511R primers (2). Isolates were screened for MDR strains using the disk diffusion assay (3). *E. hormaechei* 2B-MC1 was found to be resistant to multiple classes of antimicrobials and was selected for whole-genome sequencing. A pure culture of the strain was grown in tryptic soy broth, and DNA from the overnight culture was extracted using the DNeasy PowerFood microbial kit (Qiagen). The quality and quantity of the DNA were measured using a NanoDrop One spectrophotometer (Thermo Fisher). Libraries from the DNA sample were prepared and sequenced at a Florida State University (Florida, USA) molecular cloning facility. Libraries were sequenced using a MiSeq 2 microkit (Illumina) at 9 pM with 5% PhiX. Default parameters for all software were used during analyses unless otherwise specified. The raw Illumina sequences were quality filtered using Trimmomatic (v0.36) (4). The high-quality reads were *de novo* assembled with Velvet (1.2.10) (5) with a k-mer size of 51. We only considered contigs longer than 200 bp in computing. CheckM (v1.0.7) with “lineage_wf” workflow (6) was used to estimate the completeness of the draft genome. The most closely related complete genomes of *E. hormaechei* 2B-MC1 were identified using Microbial Genomes Atlas MiGA online tool (7) with the NCBI prokaryotic genome database. The assemblies were annotated with the Prokka (v1.11) (8) annotation pipeline. Antimicrobial-resistant gene sequences were identified with NCBI AMRFinder (9). The analysis of harbored plasmid(s) within the draft genome were detected using the PLSDB database (10) with Mash software (11). The identified plasmids were further confirmed with BLAST alignment.

A total of 4,397,799 paired-end reads were sequenced. These raw sequences were trimmed and then quality filtered (about 8% of the reads were eliminated), resulting in approximately 363,705 reads. These high-quality reads were assembled into contigs. CheckM estimated the completeness of this genome as 99.8%. This assembly produced 160 contigs and an *N*_50_ value of 82,480 bp. This draft genome was 4,661,561 bp long with a G+C content of 55.3%. Genome annotation with Prokka identified 4,336 open reading frames and 36 RNAs (1 rRNA, 34 tRNAs, and 1 transfer-messenger RNA [tmRNA]). A total of 1,567 genes were associated with KEGG pathways. The closest relatives of *E. hormaechei* 2B-MC1 found by MiGA in the database were *Enterobacter* sp. strain CRENT-193 (GenBank accession number NZ_CP024812.1) and *E. hormaechei* (NZ_CP030076) with an average nucleotide identity of 99.19%. AMRFinder identified genes and mutations that confer resistance to sulfonamides (*sul1* and *sul2*), quinolones (*qnrA1*), phenicol/quinolones (*oqxB*), trimethoprim (*dfrA23*), beta-lactamases (*bla*_ACT_), phenicols (*floR*), fosfomycin (*fosA*), tetracycline [*tet*(A)], and aminoglycosides [*aph(6)-Id* and *aph(3″)-Ib*]. The following six putative plasmids were present in the draft genome: pESBL176 (MT230180.1), pESBL31 (MT230288.1, MT230289.1, MT230292.1, and MT230305.1), pESBL87 (MT230381.1), pESBL96 (MT230424.1 and MT230426.1), unnamed4 plasmid (NZ_CP020513.1), and pKP2442_7c331 (NZ_KX434882.1).

### Data availability.

This whole-genome shotgun project has been deposited at DDBJ/ENA/GenBank under the accession number JACVEK000000000. The version described in this paper is the first version, JACVEK010000000. Raw Illumina data have been deposited in the NCBI Sequence Read Archive with the accession number SRR12586817 under the BioProject accession number PRJNA661375.
